# Predicting heterogeneous treatment effects of an Internet-based depression intervention for patients with chronic back pain: Secondary analysis of two randomized controlled trials^☆^^☆☆^^☆☆☆^

**DOI:** 10.1016/j.invent.2023.100634

**Published:** 2023-06-07

**Authors:** Mathias Harrer, David Daniel Ebert, Paula Kuper, Sarah Paganini, Sandra Schlicker, Yannik Terhorst, Benedikt Reuter, Lasse B. Sander, Harald Baumeister

**Affiliations:** a Psychology & Digital Mental Health Care, Technical University Munich, Munich, Germany; b Clinical Psychology and Psychotherapy, Friedrich-Alexander-University Erlangen-Nuremberg, Erlangen, Germany; c Clinical Psychology, Institute for Psychology, Humboldt University Berlin, Berlin, Germany; d Department of Sport Psychology, Institute of Sports and Sport Science, University of Freiburg, Freiburg, Germany; e Department of Clinical Psychology and Psychotherapy, Institute of Psychology and Education, University Ulm, Ulm, Germany; f Department Humanmedizin, Medical School Berlin, Berlin, Germany; g Department of Rehabilitation Psychology and Psychotherapy, Institute of Psychology, Albert-Ludwigs-University Freiburg, Freiburg, Germany; h Medical Psychology and Medical Sociology, Faculty of Medicine, Albert-Ludwigs University Freiburg, Freiburg, Germany

**Keywords:** Depression, Back pain, Internet-delivered CBT, Machine learning

## Abstract

**Background:**

Depression is highly prevalent among individuals with chronic back pain. Internet-based interventions can be effective in treating and preventing depression in this patient group, but it is unclear who benefits most from this intervention format.

**Method:**

In an analysis of two randomized trials (*N* = 504), we explored ways to predict heterogeneous treatment effects of an Internet-based depression intervention for patients with chronic back pain. Univariate treatment-moderator interactions were explored in a first step. Multilevel model-based recursive partitioning was then applied to develop a decision tree model predicting individualized treatment benefits.

**Results:**

The average effect on depressive symptoms was *d* = −0.43 (95 % CI: −0.68 to –0.17; 9 weeks; PHQ-9). Using univariate models, only back pain medication intake was detected as an effect moderator, predicting higher effects. More complex interactions were found using recursive partitioning, resulting in a final decision tree with six terminal nodes. The model explained a large amount of variation (bootstrap-bias-corrected *R*^2^ = 45 %), with predicted subgroup-conditional effects ranging from *d*_*i*_ = 0.24 to −1.31. External validation in a pilot trial among patients on sick leave (*N* = 76; *R*^2^ = 33 %) pointed to the transportability of the model.

**Conclusions:**

The studied intervention is effective in reducing depressive symptoms, but not among all chronic back pain patients. Predictions of the multivariate tree learning model suggest a pattern in which patients with moderate depression and relatively low pain self-efficacy benefit most, while no benefits arise when patients' self-efficacy is already high. If corroborated in further studies, the developed tree algorithm could serve as a practical decision-making tool.

## Highlights

• Effect moderators of a depression intervention for pain patients were explored.
• Multilevel tree learning was used to create a precision treatment rule.
• Predicted treatment effects varied considerably, from *d*_*i*_ = 0.24 to −1.31.
• The resulting tree model could be useful as a practical decision-making tool.

## 1 Background

Major depression is a common mental disorder. It is linked to numerous adverse outcomes including loss of quality of life, excess mortality, and large economic costs (James et al., 2018; Kessler and Bromet, 2013). Depressive disorders are highly prevalent in chronic back pain patients (Bair et al., 2003), leading to worse treatment outcomes and increased pain-related disability (IsHak et al., 2018; Linton and Bergbom, 2011; Nicholas, 2007). Interventions to treat and prevent comorbid depression are needed to improve mental health care in this patient group.

There is evidence that cognitive-behavioral therapy (CBT) is effective in chronic pain patients with comorbid depression, including back pain patients (Hoffman et al., 2007; IsHak et al., 2018). However, routine provision of specialized psychotherapeutic treatment remains rare (Bair et al., 2009; Driscoll et al., 2021). Indicated depression prevention programs are underused in the general population (Cuijpers et al., 2010), and they are also not part of routine care for pain patients.

Internet-based interventions have been discussed as a promising way to reduce the treatment gap among patients with chronic pain (Buhrman et al., 2016). Digital interventions may be helpful, for example, to provide direct access to specialized treatment formats for comorbid depression, as well as to qualified clinicians. They are also highly scalable and may allow to increase the dissemination of interventions among patients who already suffer from subthreshold depressive symptoms, and are therefore at risk of developing a full-blown comorbid depressive disorder (Ebert et al., 2017).

Internet-delivered CBT interventions have been found to be effective in chronic pain patients, both for depression and pain-related outcomes (Gandy et al., 2022). However, until 2020, only few published trials were conducted in chronic back pain patients specifically (Buhrman et al., 2004, Buhrman et al., 2011; Carpenter et al., 2012; Chiauzzi et al., 2010). Sample sizes were often small, and no trial specifically focused on patients with a comorbid depression diagnosis. Furthermore, no trial had examined if Internet-based interventions can prevent depression in chronic back pain patients.

The WARD-BP (Baumeister et al., 2021) and PROD-BP (Sander et al., 2020) trials closed this gap by examining the effect of an Internet-based intervention addressing depressive symptoms among patients with chronic back pain. Sander et al. (2020), including only patients with subthreshold depressive symptoms at baseline, found that the Internet-based intervention reduced the incidence of major depression over 12 months, as well as depressive symptoms 9 weeks post-randomization. Results of the WARD-BP trial were less conclusive. Focusing on individuals with comorbid depression, this study found effects on self-reported PHQ-9 and clinician-rated QIDS, but not on clinician-rated HAMD depressive symptom severity after 9 weeks (Baumeister et al., 2021).

These findings underline the need to identify those patients for whom Internet-based interventions to treat or prevent comorbid depression provide added benefits, and for whom they do not. Previous research has identified various factors that may influence the effectiveness of psychological interventions for chronic pain patients. For instance, Goossens et al. (2005) report that higher treatment expectancy predicted larger effects of CBT on negative affect, quality of life, motoric behavior and pain coping in patients with chronic back pain and fibromyalgia. This finding aligns with other research emphasizing the importance of expectations as both a predictor and working mechanism of treatment success in psychological interventions (Colloca and Miller, 2011; Rief et al., 2015). Similarly, it has been found that improvements in perceived pain control and self-efficacy predict better pain-related outcomes of face-to-face CBT in patients with chronic pain (Turner et al., 2007), and that self-efficacy moderates the effects of a digital pain coping skills training (Lawford et al., 2018). It has also been found that patients' commitment to using an online intervention for chronic pain moderates the effect on post-intervention pain self-efficacy (Chiauzzi et al., 2010). Lastly, Probst et al. (2019) found that psychological inflexibility, which refers to the inability to focus on the present moment and regulate behavior to achieve goals and values (Hayes et al., 2006), moderated the effects of an Internet-based Acceptance and Commitment Therapy (ACT) program on pain interference at post-test and 6-month follow-up, with lower inflexibility predicting higher treatment effects.

Despite these promising results, systematic reviews and meta-analyses have revealed significant limitations in the current literature on working mechanisms of psychological treatments for chronic pain patients. Gilpin et al. (2017), synthesizing results of *k* = 20 studies, found that findings on moderators of CBT for chronic pain were highly inconsistent and inconclusive, as well as hampered by methodological limitations. Murillo et al. (2022), examining 28 mediation and 11 moderator analyses of psychological interventions for chronic musculoskeletal pain, concluded that available evidence was conflicting and did not support a robust moderating effect for any of the examined constructs. Overall, there is still a very limited understanding about effect moderators and working mechanisms involved in psychological interventions, both digital and face-to-face (Cuijpers et al., 2019; Domhardt et al., 2021). Therefore, patients often must undergo several courses of treatment before an effective format is found (Kessler, 2018).

Several approaches have been proposed to address this issue. Cuijpers et al. (2022) suggest pooling patient data from multiple trials to explore moderators, since this increases statistical power to detect significant treatment-covariate interactions and decreases the risk of false positive findings. Additionally, there is a growing body of research that combines several putative effect moderators within multivariable prediction models, rather than evaluating them on a univariate basis (Kent et al., 2020; Kessler et al., 2019). Especially if integrated into practical “precision medicine” decision-making tools (Terhorst et al., 2023), these predictive models could greatly improve treatment selection in practice. Such tools are increasingly developed for Internet-based interventions, typically using machine learning. They are employed, for instance, to assign individual patients to formats with the largest expected benefits (Wallert et al., 2022), to identify patients with low levels of adherence (Chien et al., 2020), or to detect and assist intervention participants at risk of non-response (Forsell et al., 2019). However, we are not aware of any readily available decision-support models to determine which chronic back pain patients are expected to benefit from an Internet-based intervention for depressive symptoms as studied in the WARD and PROD-BP trials, and for whom this format is less suited.

In this study, we therefore aim to identify patient characteristics that modify the effect on post-test depressive symptoms of the Internet-based intervention for back pain patients evaluated in the WARD-BP and PROD-BP trials. Furthermore, our goal is to derive a model that allows to assess expected benefits of the intervention based on identified effect modifiers. We focus on univariate associations first, and then apply a multivariate model-based machine learning approach to construct a decision tree predicting individualized treatment effects.

## 2 Material and methods

We analyzed patient data of two large randomized controlled trials evaluating the efficacy of an Internet-based depression intervention in patients with chronic back pain (“eSano BackCare”; Baumeister et al., 2021; Sander et al., 2020). Both trials have been pre-registered in the German Clinical Trials Register (DRKS00009272, registered September 14th, 2015; DRKS00007960; registered August 12th, 2015), and have been approved by the ethics committee of the University of Freiburg (297/14, September 10th, 2014; 297/14_150513, June 23rd, 2015). All participants gave their written informed consent to participate in the studies. The secondary analyses presented in this article have been preregistered using the Open Science Framework (OSF; osf.io/sfv5a; registered February 3rd, 2022). Recommendations of the Predictive Approaches to Treatment Effect Heterogeneity (PATH; Kent et al., 2020) statement are followed where applicable.

### 2.1 Participants

For both trials, patients with chronic back pain were recruited. Recruitment took place either (i) in person, by clinic staff at discharge from eight orthopaedic rehabilitation clinics, or (ii) online, via information forms distributed by 74 German orthopaedic rehabilitation clinics after client discharge. All included participants reported persistent depressive symptoms as measured by the Patient Health Questionnaire (PHQ-9; Kroenke et al., 2001; at least two assessments with scores ≥ 5). Patients who met the DSM-5® (Falkai and Wittchen, 2018) criteria for a mild to moderate depressive episode or persistent depressive disorder were included in the WARD-BP trial (Baumeister et al., 2021). Individuals who did not meet these criteria were included in the PROD-BP trial by Sander et al. (2020).

Individuals in both studies also had to meet the following criteria to be included: (i) 18 years or older, (ii) back pain (diagnosed by the treating physician or medical records) and self-reported pain chronicity of at least six months, (iii) sufficient German language skills, (iv) Internet access. Exclusion criteria were: (i) an ongoing or planned psychotherapy, or psychotherapy within the previous six months, (ii) current suicidality or suicidal attempts within the past five years, (iii) a severe depressive episode. A more detailed description of the participants and procedures can be found elsewhere (Baumeister et al., 2021; Lin et al., 2017; Sander et al., 2017, Sander et al., 2020). For the present study, there were no additional eligibility criteria beyond the ones applied in the original trials.

### 2.2 Intervention

In both studies, participants in the intervention group received “eSano BackCare”, a guided Internet- and mobile-based intervention (IMI), as well as unrestricted access to treatment as usual (TAU). Participants allocated to control received TAU only. The “eSano BackCare” training consists of six obligatory modules and three optional modules which should be completed weekly. The intervention is based on CBT and includes psychoeducation, behavioral activation, and pain-related elements. After each session, semi-structured written feedback was delivered by trained and supervised psychologists (E-Coaches). Participants could additionally receive automated text messages which were intended to maintain motivation. E-Coaches could be contacted on demand. Booster sessions were offered two, four or six weeks after the last module. A more detailed description of the intervention can be found elsewhere (Baumeister et al., 2021; Lin et al., 2017; Sander et al., 2017, Sander et al., 2020).

### 2.3 Target outcome

The target outcome in this study, self-rated depression severity at 9-week post-test, was measured using the PHQ-9 (Kroenke et al., 2001). The PHQ-9 is a reliable and valid instrument for the criteria-based assessment of depressive symptom severity. It consists of nine items that are rated from 0 (“not at all”) to 3 (“nearly every day”). This results in a 0–27 range of total scores, where higher scores indicate higher depressive symptom severity.

### 2.4 Moderator variables

As putative moderators, we included baseline sociodemographic variables, measures of the symptom severity and health-related quality of life, as well as pain-related risk factors. The set of analyzed predictors was determined a priori (see “measured variables” section in the preregistration).

#### 2.4.1 Sociodemographic variables

As sociodemographic indicators, we selected age, gender, marital status, level of education, Internet affinity, method of recruitment, and amount of social support. Regarding baseline healthcare utilization, intake of depression and back pain medication, sick leave, and previous claim of psychotherapy for depression were included (all self-reported). Presence of a clinician-rated lifetime Structured Clinical Interview for DSM-5® (SCID; Beesdo-Baum et al., 2019) diagnosis was included as well.

#### 2.4.2 Symptom severity & quality of life

Symptom severity variables included baseline depressive symptoms, as measured by the clinician-rated Hamilton Depression Scale (HAM-D-17; Hamilton, 1960) and PHQ-9 self-report; as well as pain intensity measured by a numerical rating scale (total score range: 0–10; self-report). The Assessment of Quality of Life (AQoL-6D; Richardson et al., 2012; total score range: 0–100; self-report) was selected as a quality of life measure.

#### 2.4.3 Pain-related risk factors

As back pain-related risk factors, we included pain self-efficacy measured by the Pain Self-Efficacy Questionnaire (PSEQ; Nicholas, 2007; total score range: 0–60; self-report). In clinical settings, a PSEQ score <17 can be regarded as low, while a score >40 can be interpreted as high pain self-efficacy (Nicholas, 2007). Furthermore, we selected disability in relation with back pain, assessed by the Oswestry Disability Index (ODI; Fairbank and Pynsent, 2000; total score range: 0–100; self-report) and subjective prognosis of the working capacity, as assessed with the 3-item Subjective Prognostic Employment Scale (SPE; Mittag et al., 2003; total score range: 0–3; self-report).

### 2.5 Risk of bias assessment

Risk of bias in both studies was assessed by independent reviewers who were not involved in the original studies (see Acknowledgements). Judgements were based on criteria of the Cochrane Collaboration risk of bias assessment tool 2.0 (RoB; Sterne et al., 2019). The RoB tool 2.0 assesses risk of bias in randomized trials regarding five domains, three of which were considered in the present study (bias due to randomization procedure, deviations from intended interventions, and measurement of the outcome). The domains “missing outcome data” and “selection of reported results” were not assessed since the original study data was available.

### 2.6 Statistical analyses

All statistical analyses were conducted using the statistical computing software *R*, version 4.1.0 (R Core Team, 2021) with the significance level set to α = 0.05. Code used for the analyses has been made publicly available in an OSF repository (osf.io/xz5nj).

#### 2.6.1 Missing data handling

Our analyses follow the intention-to-treat principle, meaning that all participants who were randomized to the treatment conditions were subsequently analyzed. Missing data at baseline and post-assessment were estimated via Multivariate Imputation by Chained Equations (MICE; fully conditional specification) under a missing at random (MAR) assumption. To develop the imputation model, we first examined missingness patterns and predictors of missingness in the target variable (Carpenter and Smuk, 2021; see S1 and S2 in the Supplement). We then included baseline covariates (demographic variables, treatment history, risk factors, pain- and symptom-related measures) as auxiliary variables into the imputation model. Variables were removed from the predictor matrix when their correlation with the imputed variable was below *r* = 0.05. The final imputation predictor matrix is presented in S3 in the Supplement.

Imputations were generated separately for the intervention and control group (groupwise imputation). Since the substantive analysis models employed in this study are hierarchical (patients-in-trials), a multilevel imputation model was developed. Numeric variables were estimated using a two-level normal model (Schafer and Yucel, 2002), while predictive mean matching (PMM) was used for categorical values. The imputation model was implemented using the R packages “*mice*” (Van Buuren and Groothuis-Oudshoorn, 2011) and “*miceadds*” (Robitzsch and Grund, 2022). Due to the small number of clusters (viz. trials), we used a maximum penalized likelihood (MPL)-based approach to estimate the heterogeneity variances *τ*^2^. This method can be seen as equivalent to estimating *τ*^2^ by its posterior mode conditional on a weakly informative Wishart prior. This estimation method was chosen because non-zero heterogeneity variances were assumed to be unlikely, with the MPL approach allowing to a priori rule out boundary fits during imputation. A total of *m* = 50 imputation sets was generated. Analyses were conducted within the multiply imputed data, and parameters were pooled using Rubin's rules (Barnard and Rubin, 1999). The Rubin combination rules are not directly applicable to nonparametric approaches such as random forests and model-based recursive partitioning. These algorithms were therefore applied in an aggregated data set based on the multiply imputed data.

#### 2.6.2 Average treatment effect

A one-stage meta-analysis model was used to calculate the overall effect of the intervention on depression severity at post-assessment. To circumvent issues of singularity and downward-biased standard errors, the same “pseudo-Bayesian” MPL estimation framework as used in the imputation model was applied. For the random effects variance-covariance matrix, we set a Wishart prior with ν=4 and an identity matrix multiplied by 0.05 on the scale parameter to generate a maximum a posteriori estimate in all analyses. This prior is designed to avoid boundary fits while still being largely uninformative. In the model formula, depression severity at post-test was regressed on the treatment indicator while adjusting for baseline depression symptom severity, which was stratified by trial. Additionally, we also calculated the rates of participants achieving reliable improvement in depressive symptom severity at post-test based on the reliable change index (RCI; Jacobson and Truax, 1992), and tested if they differed between groups.

#### 2.6.3 Univariate treatment-moderator interactions

To investigate potential moderator variables, we first examined individual treatment-covariate interactions. For each moderator variable, a separate linear mixed model with random trial intercepts and trial-specific group slopes was used, which incorporated an interaction term with the treatment indicator. To fit the models, we employed the same MPL-based estimation framework implemented in the “*blme*” package (Chung et al., 2013) that was also used to impute missing values (Section 2.6.1) and calculate the average treatment effect (Section 2.6.2). To compare the results to a model without random effects, we also conducted a sensitivity analysis in which treatment-covariate interactions were examined using OLS regression models. The intercept of these sensitivity analysis models was stratified by trial, and cluster-robust variance estimation was used to test the interaction (“CR2” estimator by Pustejovsky and Tipton, 2018). The exact specification of the main and sensitivity analysis models is described in S4 in the Supplement. Additionally, we also conducted a change score analysis in which continuous moderator variable scores were grouped by percentile ranges (<20th, <40th, 40th to 60th, >60th, >80th percentile). In this analysis, means and change scores as well as their standard error were calculated for each percentile group or moderator category. This was done to examine treatment effect patterns across different levels of the analyzed moderators. Analyses were performed in the multiply imputed data. As a further sensitivity analysis, we also examined the results of both models when only complete cases were considered.

#### 2.6.4 Multivariate treatment-moderator interactions

In a next step, the putative moderators were assessed for their relevance in higher-order interaction analyses by calculating variable permutation importance indices. This was achieved using the model-based random forest methodology proposed by Garge et al. (2013). In this approach, multiple (in our case *n* = 300) model-based trees are constructed. One-third of the included moderators are randomly selected as splitting variables in each tree, leading to more stable and less sample-specific predictions. Nodes in each tree were considered for further splitting if the Bonferroni-corrected *p*-value of any partitioning variable in that node fell below α = 0.05, allowing a minimum of 20 observations in each node. The underlying objective was to preselect a more parsimonious set of variables for the subsequent multivariate analysis (Genuer et al., 2010). In the node model, symptoms of depression at post-test were regressed on the treatment condition and all potential moderators were included as partitioning variables on their raw scale. Variables with a positive importance index and/or significant moderators of the univariate moderation analysis were included as partitioning variables in the subsequent analysis.

Model-based recursive partitioning (MOB) trees as introduced by Zeileis et al. (2008) were then used to explore more complex treatment-moderator interactions. MOB trees allow to apply decision tree learning, a common machine learning method, to parametric models fitted using *M*-type estimators (e.g. OLS or maximum likelihood; Fokkema et al., 2021; Zeileis et al., 2008) assuming that a single global model does not fit the data well. In our case, MOB trees were fitted using a multilevel linear model in the tree nodes, in which we also used the “pseudo-Bayesian” estimation framework as employed in the models described above. As a stopping rule, the significance level for parameter stability tests was set to α = 0.05 and *p*-values were Bonferroni-corrected (“pre-pruning”). Cohen's *d* was calculated as a standardized measure of the “individualized” conditional average treatment effect (CATE) prediction in each terminal node subgroup. We also calculated the rates of reliable improvement and calculated effect sizes in the complete case subsample in each terminal node as a sensitivity analysis. Furthermore, to explore the robustness of our results, we additionally fitted a multivariable linear mixed model with a LASSO penalty (Groll and Tutz, 2014). This model included all putative moderator variables that were also considered for the tree model, which were included both as simple “prognostic” and “prescriptive” treatment-covariate interaction terms. The penalty parameter *λ* of this model, which controls the shrinkage applied to estimated parameters, was determined using the Bayesian Information Criterion (BIC).

The optimism-corrected performance of the resulting tree was calculated using the bootstrap bias correction approach by Harrell Jr et al. (1996), with *B* = 1000 bootstrap samples. To explore the transportability to a plausibly related setting (Justice et al., 1999), we also calculated the decision tree's performance when applied to unseen data of the “Get.Back” pilot trial by Schlicker et al. (2020; *n*=76). Using a similar design as the WARD-BP and PROD-BP studies, this trial focused exclusively on patients with chronic back pain who are currently on sick leave. The intervention employed in “Get.Back” was also based on the “eSano BackCare” training, with some minor adaptions to suit people on current sick leave. Further study and participant characteristics of this trial are provided in the main outcome publication (Schlicker et al., 2020).

## 3 Results

**Fig. 1:**
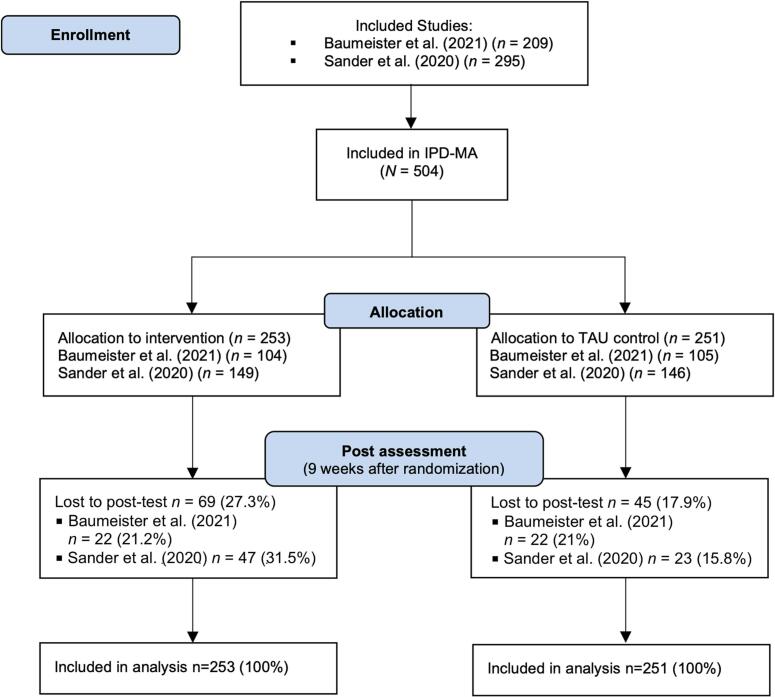
Combined flow chart of the included WARD-BP (Baumeister et al., 2021) and PROD-BP (Sander et al., 2020) trials.

An overview of participant enrolment and loss to follow-up is depicted in Fig. 1. Merging the data of both trials resulted in a final sample of *N* = 504 participants. A total of *n* = 253 individuals were allocated to the intervention group (IG), and *n* = 251 individuals to the control group (CG). An overview of the risk of bias assessment for both studies is depicted in Fig. S5 in the Supplement. Participant characteristics at baseline are summarized in Table 1. The proportion of women in the sample was 61.31 %, and the average age at baseline was 51.59 (*SD* = 8.55). The mean level of PHQ-9 depressive symptoms at baseline was *M* = 10.06 (*SD* = 4.55; range 0–25), indicating moderate depression across both trials.

**Table 1:** Participant characteristics at baseline.

Characteristic	All participants (*N* = 504)	Control (*N* = 251)	Intervention (*N* = 253)
Recruitment method			
On-site, *n* (%)	356 (70.63)	175 (69.72)	181 (71.54)
Online, *n* (%)	148 (29.37)	76 (30.28)	72 (28.46)
Socio-demographics			
Age, *M* (*SD*)	51.59 (8.55)	52.1 (8.19)	51.09 (8.88)
Gender, female, *n* (%)	309 (61.31)	160 (63.75)	149 (58.89)
In a relationship, yes, *n* (%)	365 (72.42)	185 (73.71)	180 (71.15)
Children, yes, *n* (%)	402 (79.76)	202 (80.48)	200 (79.05)
Current sick leave, yes, *n* (%)	351 (69.64)	173 (68.92)	178 (70.36)
Lifetime SCID diagnosis, yes, *n* (%)	219 (43.45)	110 (43.82)	109 (43.08)
Marital status			
Single, *n* (%)	50 (9.92)	23 (9.16)	27 (10.67)
Relationship/married, *n* (%)	365 (72.42)	185 (73.71)	180 (71.15)
Divorced/separated, *n* (%)	74 (14.68)	35 (13.94)	39 (15.42)
Widowed, *n* (%)	15 (2.98)	8 (3.19)	7 (2.77)
Educational level			
No formal education (completed), *n* (%)	76 (15.08)	28 (11.16)	48 (18.97)
Up to high school (7–9 years), *n* (%)	164 (32.54)	87 (34.66)	77 (30.43)
High school education (12–13 years), *n* (%)	201 (39.88)	105 (41.83)	96 (37.94)
After high school, *n* (%)	39 (7.74)	20 (7.97)	19 (7.51)
Social support			
Little social support, *n* (%)	142 (28.17)	75 (29.88)	67 (26.48)
Sufficient social support, *n* (%)	146 (28.07)	65 (25.9)	81 (32.01)
Good social support, *n* (%)	147 (29.17)	74 (29.48)	73 (28.85)
Very good social support, *n* (%)	51 (10.12)	28 (11.16)	23 (9.09)
Prior treatment experience			
Previous psychotherapy experience, yes, *n* (%)	182 (36.11)	91 (36.25)	91 (35.97)
Previous depression medication, yes, *n* (%)	142 (28.17)	71 (28.29)	71 (28.06)
Previous back pain medication, yes, *n* (%)	322 (63.89)	160 (63.75)	162 (64.03)

### 3.1 Average treatment effect

At post-test, the mean change in PHQ-9 scores from baseline was −0.97 points in the control group (95 % CI: −1.51 to −0.44), and − 2.94 points (95 % CI: −3.51 to −2.37) in the intervention group. The difference between IG and CG on PHQ-9 at post-test was significant (*β* = −2.07 95 % CI: −3.31 to −0.83; *t* = −3.29, *p* < 0.01), resulting in an estimated effect of *d* = −0.43 (95 % CI: −0.68 to −0.17) favoring the intervention. The estimated between-study heterogeneity variance of the treatment effect was $\hat{\tau }$^2^ = 0.48. Similar results emerged in a sensitivity analysis focusing only on participants with complete baseline and post-test PHQ-9 data (*β* = −2.11, *t* = −3.61; *d* = −0.44, 95 % CI: −0.67 to −0.20, $\hat{\tau }$^2^ = 0.41). More participants in the intervention group (*n* = 62; 25 %) achieved reliable improvement compared to the control group (*n* = 28; 11 %; *F*_1,1515.47_ = 12.36; *p* < 0.001; see S6 in the Supplement).

### 3.2 Moderator analysis

#### 3.2.1 Univariate treatment-moderator interactions

Results of the univariate analysis of treatment-covariate interactions are displayed in Table 2. Back pain medication was found to be a significant moderator (*p* < 0.05). Higher effects were predicted for patients who take back pain medication (*d* = −0.57; 95 % CI: −0.88 to −0.26), and lower effects for those whose do not (*d* = −0.17; 95 % CI: −0.49 to 0.15). All other first-order interaction effects were not significant (*p* ≥ 0.05). The complete case analysis mirrored these findings (see S7 in the Supplement).

**Table 2:** Regression coefficients of univariate moderator analyses.

Baseline variable	Treatment-covariate interaction					
	$\hat{\beta }$	$\sqrt{{\hat{V}}_{\beta }}$	*t*	*p*(<|*t*|)	${\hat{\tau }}_{\text{Intercept}}^{2}$	${\hat{\tau }}_{\text{Slope}}^{2}$
Socio-demographics						
Age	0.050	0.453	0.110	0.913	2.233	0.601
Gender, female	−0.665	0.915	−0.727	0.467	2.238	0.597
Relationship, yes	−0.886	0.875	−1.012	0.312	2.220	0.612
Education, >13 years	−0.544	0.921	−0.591	0.555	2.286	0.632
Children, yes	−0.661	0.890	−0.743	0.458	2.225	0.593
Lifetime SCID diagnosis, yes	−0.737	1.484	−0.497	0.619	1.019	0.905
Previous psychotherapy, yes	0.031	0.925	0.034	0.973	2.164	0.610
Previous depression medication, yes	0.503	0.927	0.543	0.587	2.191	0.646
Previous back pain medication, yes	−1.945	0.886	−2.194	0.028	2.206	0.612
Sick leave, yes	−1.320	1.007	−1.311	0.191	2.056	0.555
Social support, (very) good	1.012	0.889	1.139	0.255	2.134	0.586
Internet affinity (IAS)	−0.006	0.458	−0.013	0.990	2.164	0.596
Recruitment, online	1.028	0.891	1.155	0.248	2.304	0.636
Symptom severity						
Depressive symptom severity (PHQ-9)	−0.763	0.413	−1.847	0.065	0.444	0.348
Depressive symptom severity (HAM-D-17)	−0.752	0.480	−1.568	0.117	0.359	0.491
Pain intensity (NRS)	−0.423	0.444	−0.952	0.341	1.941	0.616
Quality of life (AQoL-6D)	−0.477	0.430	−1.108	0.268	0.390	0.557
Pain-related risk factors						
Pain self-efficacy (PSEQ)	0.072	0.438	0.164	0.870	1.376	0.904
Subjective employment forecast (SPE)	−0.326	0.449	−0.726	0.468	1.925	0.559
Pain-related disability (ODI)	−0.256	0.429	−0.598	0.550	1.408	0.634

Note. A Wishart prior was used for the covariance-variance matrix of the random effects. AQoL-6D: Assessment of Quality of Life, HAM-D-17: Hamilton Depression Rating Scale, IAS: Internet Affinity Scale; NRS: numerical rating scale, ODI: Oswestry Disability Scale, PHQ-9: Patient Health Questionnaire, PSEQ: Pain self-efficacy questionnaire, SPE: Subjective Prognostic Employment Scale.

Results of the sensitivity analysis employing OLS regression are presented in S8 in the Supplement. Findings were comparable to the ones of the main model analysis, except that baseline PHQ-9 scores were additionally found as a significant moderator (*p* = 0.035), whereby higher baseline symptom severity predicted higher treatment effects (*β* = −0.779). The same pattern of findings also emerged in the complete case sample (see S9 in the Supplement).

Results of the change score analysis are presented in S10 in the Supplement. Findings closely mirrored the ones of the main analysis. Only previous back pain medication emerged as a significant predictor of differential treatment effects (*t* = 6.606, *p* = 0.01), whereby patients with medication intake experienced an additional average decrease of 2.05 points on the PHQ-9 compared to patients without pain medication. A plot of the control and intervention group change in PHQ-9 scores for different percentile groups based on baseline PHQ-9 is provided in S11 in the Supplement. Examining the point estimates alone revealed that higher baseline PHQ-9 scores were associated with higher decreases in PHQ-9 scores (<20th percentile: −1.46, <40th: −1.38, 40th to 60th: −1.40, >60th: −2.88, >80th: −3.26). However, we did not find that this difference was significant overall (*p* = 0.319), nor did we find that any direct comparisons of change scores between percentile groups were significant (all *p* ≥ 0.05).

#### 3.2.2 Predictor selection for the multivariate analysis

Fifteen of all 20 putative moderators had positive permutation importance values and were thus selected as partitioning variables for further analyses (see Fig. S13 in the Supplement). Age, gender, sick leave, previous depression medication and previous claim of psychotherapy had negative importance values and were therefore excluded. Variable importance was highest for baseline symptom severity assessed via PHQ-9 and HAM-D-17, health-related quality of life, pain self-efficacy and disability, with values ranging from 6.75 to 0.32. A similar finding emerged when only complete cases were considered (see S14 in the Supplement). In S12 in the Supplement, we display the intercorrelation of all putative moderators as well as their predictive association with the target outcome. Intercorrelations ranged from |*r*| = 0 to 0.74, with no indications of problematic levels of multicollinearity (all variance inflation factors <3).

#### 3.2.3 Multilevel model-based recursive partitioning

**Fig. 2:**
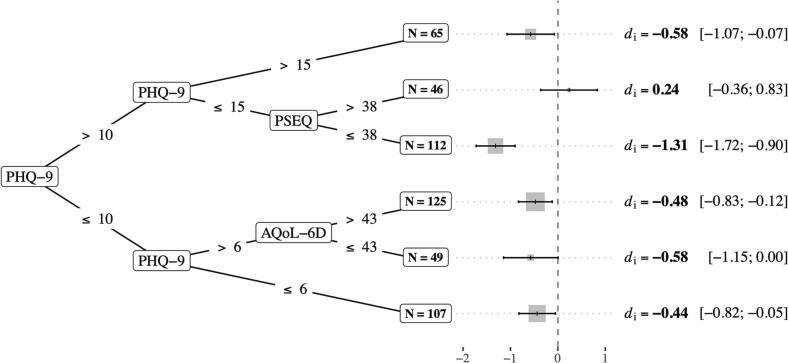
Final model-based decision tree.

Fig. 2 displays the final multilevel model-based tree and effect sizes in the terminal nodes. Six subgroups with differential treatment effects were identified. The data first partitioned individuals based on their PHQ-9 score using a cut-off value of 10 (*p* < 0.001), which roughly coincides with the sample mean. The subgroup with values ≤ 10 was further split based on a PHQ-9 score of 6, which lies approximately one standard deviation below the sample mean (*p* < 0.001). The resulting subgroup with PHQ-9 > 6 was then split into two groups again based on individuals' self-reported health-related quality of life, using an AQoL-6D score cut-point of 43. Overall, estimated subgroup-conditional effects in the terminal nodes were very similar for all PHQ-9 ≤ 10 pathways (*n* = 281; 55.8 %), and comparable to the average treatment effect (*d*_i_ = −0.58 to −0.44 versus *d* = −0.43).

In contrast, effect estimates varied greatly in patients with baseline PHQ-9 values >10. In the subgroup of *n* = 112 (22.2 %) patients with both PHQ-9 scores ≤15 and low-to-moderate baseline pain self-efficacy (PSEQ ≤ 38), estimated intervention effects were much higher than the average effect (*d*_*i*_ = −1.31; 95 % CI: −1.72 to −0.90), while a non-significant negative effect was found in individuals with high pain self-efficacy (PSEQ>38; *d*_*i*_ = 0.24; 95 % CI: −0.36 to 0.83; *n* = 46; 9.1 %). The model predicted an effect of *d*_*i*_ = −0.58 (95 % CI: −1.07 to −0.07) for individuals with very high depressive symptom severity (PHQ-9 > 15; *n* = 65; 12.9 %). Similar findings emerged when only complete cases were considered (see S15 in the Supplement). Results on reliable improvement in the terminal nodes mirrored the estimated effect sizes. In the 10 < PHQ-9 ≤ 15 subgroup, 75 % (*n* = 45) of all intervention group participants with low pain self-efficacy showed reliable improvement, significantly more than in the control group (35 %, *n* = 18; *F*_1,681.39_ = 11.729, *p* < 0.001). This contrasts with IG patients with high pain self-efficacy, where improvement rates were considerably lower (59 %, *n* = 10) and did not differ significantly from the control group (65 %, *n* = 17; *F*_1,47__,__174.22_ = 0.006, *p* = 0.936). Comprehensive results are presented in S16 in the Supplement.

The apparent performance of the decision tree model in predicting PHQ-9 depressive symptom severity at post-test was *R*^2^_app_ = 52 %. A roughly comparable amount of variation was explained by the multivariable LASSO model (*R*^2^_app_ = 47 %), in which the PSEQ also emerged as a main predictor of differential treatment effects (β=0.036, *p* < 0.001; see S17 in the Supplement for all estimated parameters). The bootstrap bias corrected performance of the tree model was *R*^2^_adj_ = 45 %. External validation of the model in the “Get.Back” pilot trial resulted in a predictive performance of *R*^2^_adj_ = 33 %.

## 4 Discussion

In this study, we investigated treatment effect moderators of an Internet-based depression intervention for patients with chronic back pain. Data of two randomized controlled trials, conducted in chronic back pain patients with and without comorbid major depression, were included. One-stage meta-analysis revealed a pooled effect of *d* = −0.43 (95 % CI: −0.68 to −0.17) on depressive symptom severity at post-test (9 weeks). This result is comparable to meta-analytic effects found in Internet-delivered CBT interventions across different types of chronic pain (*g* = −0.43; Gandy et al., 2022). Only back pain medication intake was found to be a significant moderator of treatment effects, predicting higher effects ($\hat{\beta }$ = − 1.95). Based on this estimate, intervention participants with pain medication intake are expected to show an average decrease of roughly 2 points on the PHQ-9 compared to those without pain medication intake.

In the main mixed model, depressive symptom severity was not a significant predictor of differential treatment effects. This is a surprising finding, given that this variable is frequently reported as an effect moderator in Internet-based interventions (Karyotaki et al., 2017; Reins et al., 2021). However, we did find that higher baseline PHQ-9 scores predicted larger treatment effects in our sensitivity analysis using OLS regression. Notably, the point estimates of both models largely agreed (main model: *β* = −0.763; sensitivity analysis: *β* = −0.799). In the additional change score analysis, we also found that benefits were larger in patients with high baseline symptom severity when looking at the point estimates alone, but this relationship also did not reach conventional levels of significance. Overall, this suggests that the divergent findings could be explained by statistical power, and that baseline symptom severity may very likely also be an effect modifier.

A clearer picture emerged when we investigated higher-order interactions using multilevel model-based decision tree learning. We found that baseline depressive symptom severity was indeed involved in explaining heterogeneity of treatment effects when examined in combination with other baseline indicators. This finding was most pronounced among individuals with moderate depressive symptoms (viz. PHQ-9 scores between 11 and 15), where low-to-moderate pain self-efficacy predicted very large treatment effects (*d*_i_ = −1.31). A non-significant negative effect was found in patients with high pain self-efficacy (*d*_i_ = 0.24). In all other identified groups, predicted treatment benefits (*d*_i_ = −0.58 to −0.44) remained close to the pooled effect established via one-stage meta-analysis (*d* = −0.43). Additionally, we found that the tree model still explained about one third of the outcome variation when applied to unseen data of the pilot trial by Schlicker et al. (2020), which provided the studied intervention to chronic back pain patients who are currently on sick leave. This suggests that the model predictions could be transportable across plausibly related contexts.

These findings provide evidence that a decision-tree model can be used to explain and predict treatment effect heterogeneity of the studied intervention. It provides a simple decision-support algorithm that allows to determine if predicted benefits are in line with the average treatment effect, or if substantially higher or lower effects are to be expected. A maximum of two self-report questionnaire scores is needed to make individualized predictions, suggesting that such an algorithm is economical enough to be useful in practice. If corroborated in further studies, the tree model could be implemented as a simple screening tool within the Internet-based intervention. This would allow to identify patients for whom the program is not expected to have any meaningful effects, and to initiate a more intensive evidence-based treatment (e.g., face-to-face CBT for chronic pain) instead. Previous studies have determined that an effect size of *d* = −0.24 represents the minimally important difference that can be perceived as beneficial from a patient perspective (Cuijpers et al., 2014). Using this criterion, only patients in one tree node would be assigned to a different treatment, representing 9.1 % of the studied population. Alternatively, one may also employ a more conservative assignment rule, in which only patients in the tree node with substantial expected effects (*d* = −1.31; 22.2 % of the study population) receive the Internet-based intervention. Future studies may investigate which of these assignment regimens is the most useful to maximize clinical benefits across all patients.

The tree structure itself also points to a possible working mechanism of the intervention: in patients with moderate depressive symptoms, the intervention may increase the confidence in being able to perform activities despite chronic pain (viz., pain-related self-efficacy; Turk and Okifuji, 2002), explaining the substantial improvements in individuals with lower PSEQ values at baseline. This may also account for the null effect in individuals who already display high pain self-efficacy (i.e., PSEQ>38). More research is needed to shed light on this hypothesis.

Overall, our findings are in line with previous studies that support the potential of multilevel model-based trees in detecting differential treatment effects of psychological interventions. Fokkema et al. (2018), for example, used multilevel model trees to identify differential effects of CBT versus pharmacotherapy, yielding a correlation between observed and predicted scores of *r* = 0.272. Driessen et al. (2022) used a similar methodology to examine differential effects of short-term psychodynamic therapy and antidepressants to antidepressants alone and found correlations of *r* = 0.077 to 0.465 for predicting post-test depression scores.

This study has some limitations. Although we pooled data of two trials for this analysis, more data would have been valuable. Univariate moderator analyses typically require much more statistical power than analyses of the average treatment effect, especially if interactions are subtle (Brookes et al., 2004; Cuijpers et al., 2022). In this context, it should be reiterated that “absence of evidence is not evidence of absence”. It is very much possible that more of the variables examined in this study do have a moderating effect on outcomes, but that larger datasets are needed to confirm this. In a similar way, only a limited range of baseline covariates assessed in the original studies could be considered for our moderator analyses. That means there may be more clinical or psychological markers that moderate the efficacy of the intervention in practice, but were not part of this evaluation. Large-scale IPD meta-analyses of Internet-based depression interventions for patients with chronic back pain may be helpful in the future to ascertain which characteristics have a clinically relevant impact on treatment effects, although this may come at the cost of higher between-study heterogeneity. Similarly, recursive partitioning as applied in our multivariate analysis comes with a large number of effective degrees of freedom (Ye, 1998), which can lead to overfitting and instable tree structures in small samples (Austin et al., 2010; Steyerberg, 2019). In this study, we used a relatively conservative pre-pruning scheme, adjusted for model optimism using bootstrap bias correction (which is the approach recommended in the methodological literature in contrast to, e.g., train-test-splits; Riley et al., 2021, chap. 17.7.1.7; Steyerberg, 2019), and examined the model performance in an external dataset. Nevertheless, our results are exploratory and should be interpreted cautiously. From a model development perspective, the “Get.Back” pilot trial used for external validation is still relatively small (*N* = 76), and only represents one plausible intervention context. Further validation studies are needed to confirm that the tree structure established in this study is indeed robust and clinically useful. Furthermore, we only considered a narrow range of algorithms in this study, and it could be explored if other modelling approaches provide additional benefits. Another limitation is that, to allow for joint analyses, we used self-reported PHQ-9 scores at 9-week post-test as the target outcome. At the same measurement point, observer-masked clinician-rated depression obtained via the HAM-D-17 was used as the primary outcome in Baumeister et al. (2021), but this outcome was not measured in both trials at post-test. Using HAMD-D-17 scores as target variable could have been valuable, particularly because no significant effect was found in the trial by Baumeister and colleagues. Lastly, our analyses focused on predictors of depressive symptom severity at post-test, since this is the outcome targeted by the studied intervention. However, we did not assess differential effects on other potentially relevant outcomes in pain management, such as pain interference or quality of life. These target variables could be considered in future research to further supplement the findings in this study.

In sum, our findings suggest that an Internet-based intervention can be effective in reducing depressive symptoms among chronic back pain patients. Additional intake of back pain medication was associated with higher effects. Results of a decision tree model point to a more complex interaction pattern, whereby low-to-moderate pain self-efficacy predicts very high treatment effects in patients with moderate depression, but no benefits if patients already experience high self-efficacy at baseline.

## Funding

MH is supported by a fellowship of the Bavarian Research Institute for Digital Transformation (BIDT), an institute of the 10.13039/501100007306Bavarian Academy of Sciences and Humanities. WARD-BP was funded by the German 10.13039/501100002347Federal Ministry of Education and Research (project “effectiveness of a guided web-based intervention for depression in back pain rehabilitation aftercare,” grant No. 01GY1330A; 01GY1330B). PROD-BP was additionally funded by the 10.13039/501100001659German Research Foundation (DFG, BA3407/5-1).

## CRediT authorship contribution statement

HB, LBS, and DDE developed the concept of the present study. HB, LBS, SP and SaS contributed to the development of the Internet-based interventions, coordinated the clinical trials, and collected data. MH and PK performed the analyses. MH, DDE, and PK drafted a first version of the manuscript. All authors contributed to the further development of the manuscript, as well as read and approved the final manuscript.

## Declaration of competing interest

DDE reports to have received consultancy fees or served in the scientific advisory board from several companies such as Novartis, Sanofi, Lantern, Schön Kliniken, Minddistrict, and German health insurance companies (BARMER, Techniker Krankenkasse). DDE and MH are stakeholders of the Institute for Health Trainings Online (GET.ON/HelloBetter), which aims to implement scientific findings related to digital health interventions into routine care. HB reports to have received consultancy fees, fees for lectures or workshops from chambers of psychotherapists and training institutes for psychotherapists and license fees for an Internet intervention.

## Data Availability

Original data included in this study are available upon reasonable request.

## Appendix A Supplementary data

Supplementary material Image 1
