# Supplementary material for: Predicting heterogeneous treatment effects of an Internet-based depression intervention for patients with chronic back pain: Secondary analysis of two randomized controlled trials
Source: Internet Interv. 2023 Jun 7;33:100634. doi: 10.1016/j.invent.2023.100634 (PMC10457531; doi:10.1016/j.invent.2023.100634)
Supplement: Supplementary file 1 — Supplementary material [file mmc1.pdf]

PREDICTING HETEROGENEOUS TREATMENT EFFECTS OF AN INTERNET-BASED DEPRESSION INTERVENTION FOR PATIENTS WITH CHRONIC BACK PAIN: SECONDARY ANALYSIS OF TWO RANDOMIZED CONTROLLED TRIALS

Supplement

Table of Contents

S1. Missingness patterns 2
S2. Predictors of missingness. 2
S3. Imputation predictor matrix. 3
S4. Model equations (univariate moderator analysis). 4
S5. Risk of Bias summary. 5
S6. Effects on reliable change. 5
S7. Regression coefficients of univariate moderator analyses (main model; complete cases only). . 5
S8. Regression coefficients of univariate moderator analyses (OLS regression). 6
S9. Regression coefficients of univariate moderator analyses (OLS regression; complete cases). ... 7
S10. Moderator-specific marginal means and change scores. 8
S11. PHQ-9 symptom changes in baseline PHQ-9 percentile groups. 12
S12. Correlation matrix of moderator variables 13
S13. Variable permutation importance (model-based random forest) 14
S14. Variable importances (complete cases only). 14
S15. Model-based recursive partitioning (complete cases only; *n*=382) 14
S16. Reliable change in terminal tree nodes. 15
S17. Non-zero parameters of the linear mixed model with $\ell 1$-estimation (glmmLasso) 15

S1. Missingness patterns.

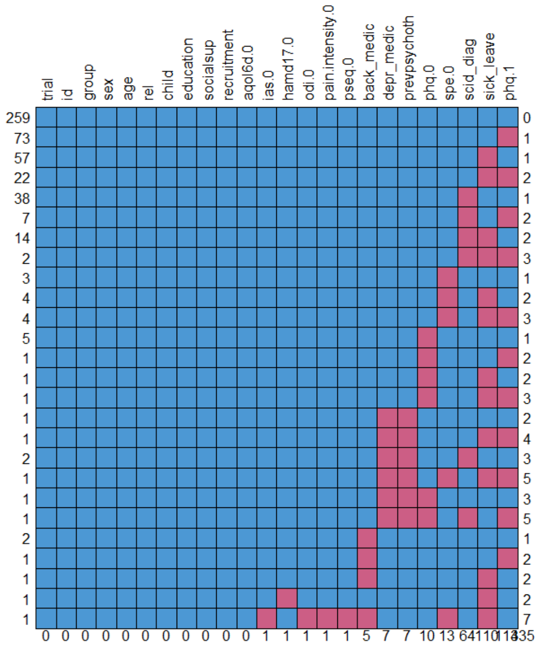

Interpretation. The most common missingness patterns (apart from complete data) are missing data in the outcome only, missing data in sick leave only and missing data in the SCID lifetime diagnosis for depression variable only.

S2. Predictors of missingness.

Baseline Variable	Prediction of missingness indicator							
	$\hat{\beta}$	*OR*	$\sqrt{\hat{V}_{\beta}}$	*Z*	$p(<\|t\|)$	$\hat{\tau}^2_{\text{Intercept}}$	$\hat{\tau}^2_{\text{Slope}}$	*n*
Group	0.529	1.697	0.231	2.291	0.022	0.006	0.012	504
**Socio-demographics**								
Age	0.004	1.004	0.013	0.331	0.741	0.010	0.000	504
Gender, female	0.186	1.204	0.226	0.821	0.412	0.007	0.008	504
Relationship, yes	0.019	1.019	0.248	0.075	0.940	0.007	0.008	504
Education, >13 years	-0.033	0.968	0.335	-0.098	0.922	0.006	0.009	504
Children, yes	-0.132	0.876	0.267	-0.495	0.620	0.007	0.007	504
Lifetime SCID diagnosis, yes	-0.042	0.959	0.269	-0.155	0.877	0.006	0.008	440
Previous psychotherapy, yes	0.128	1.137	0.233	0.550	0.582	0.008	0.008	497
Previous depression medication, yes	-0.310	0.733	0.257	-1.208	0.227	0.006	0.009	497
Previous back pain medication, yes	0.276	1.318	0.238	1.159	0.246	0.008	0.008	499
Sick leave, yes	-0.152	0.859	0.265	-0.571	0.568	0.008	0.008	394
Social support, (very) good	-0.238	0.789	0.232	-1.025	0.305	0.007	0.008	504
Internet affinity (IAS)	0.004	1.004	0.030	0.122	0.903	0.010	0.000	503
Recruitment, online	-0.371	0.690	0.255	-1.452	0.146	0.006	0.009	504

**Symptom severity**								
Depressive symptom severity (PHQ-9)	-0.006	0.994	0.028	-0.208	0.835	0.009	0.000	494
Depressive symptom severity (HAM-D-17)	0.015	1.015	0.025	0.585	0.559	0.009	0.000	503
Pain intensity (NRS)	-0.132	0.876	0.160	-0.827	0.408	0.008	0.004	503
Quality of life (AQoL-6D)	-0.002	0.998	0.013	-0.117	0.907	0.010	0.000	504
**Pain-related risk factors**								
Pain self-efficacy (PSEQ)	-0.004	0.996	0.010	-0.424	0.672	0.009	0.000	503
Subjective employment forecast (SPE)	-0.017	0.983	0.109	-0.159	0.874	0.007	0.004	491
Pain-related disability (ODI)	-0.003	0.997	0.009	-0.307	0.759	0.010	0.000	503

Note. Multilevel logistic regression results with the missingness indicator (1= missing, 0= not missing) of the primary outcome (PHQ-9 at post-assessment) as dependent variable, based on the unimputed dataset

S3. Imputation predictor matrix.

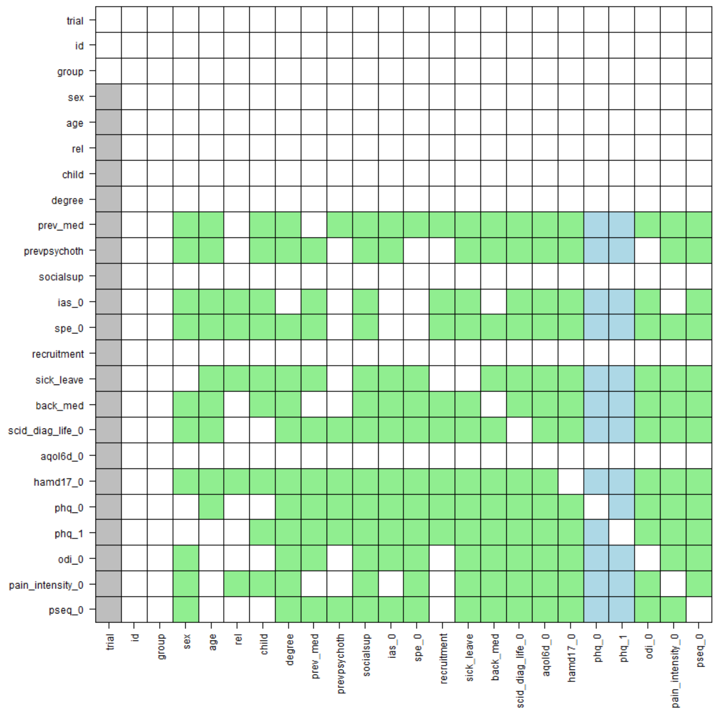

Note. Variables in the rows are predicted by variables in the columns. Missing values were generated using "by group"-imputation using multilevel models with trial as the cluster variable. Depressive symptom severity (PHQ-9) at baseline and post-assessment was modeled as both a fixed and random effect (blue); variables shown in green were modeled as simple linear predictors. A minimal correlation of r=0.05 was required for a variable to be included as a predictor.

S4. Model equations (univariate moderator analysis).

Main Model (Linear Mixed-Effects Model)

The following formula was used in the main model to test treatment-covariate interactions:

$$y_{ik} = \alpha + \delta T_{ik} + \beta x_{ik} + \gamma x_{ik} T_{ik} + u_{\alpha,k} + u_{\delta,k} T_{ik} + \varepsilon_{ik}$$

$$\begin{bmatrix} u_{\alpha,k} \\ u_{\delta,k} \end{bmatrix} \sim \mathcal{N}(\mathbf{0}, \mathbf{D}) \quad \mathbf{D} = \begin{bmatrix} \tau_{u\alpha}^2 & \rho\tau_{u\alpha}\tau_{u\delta} \\ \rho\tau_{u\alpha}\tau_{u\delta} & \tau_{u\delta}^2 \end{bmatrix}$$

Where:

- y_{ik} is the PHQ-9 post-test score of individual i in trial k;
- α is the fixed intercept;
- $u_{\alpha,k}$ is the random intercept of trial k, capturing the between-study random effects variance $\tau_{u\alpha}^2$;
- T_{ik} is the treatment assignment of individual i in trial k (0 = control, 1 = intervention), and δ the "overall" treatment effect;
- $u_{\delta,k}$ is the random group slope of trial k, capturing the heterogeneity variance of treatment effects across trials $\tau_{u\delta}^2$;
- x_{ik} is the value of individual i in trial k on the tested baseline covariate x, with β being the associated regression weight;
- γ captures the treatment-covariate interaction (i.e., moderation) of δ for varying values of x;
- ε_{ik} is the prediction error of individual i in trial k.

Given the low number of clusters, models were fitted using a maximum a posteriori estimation in a Bayesian setting as implemented in the *blme* package in R. For the random effects variance-covariance matrix $\mathbf{D}$, we set a Wishart prior with ν=4 and an identity matrix multiplied by 0.05 on the scale parameter.

OLS Regression Model (Sensitivity Analysis)

To test treatment-covariate interactions using a model without random effects, the following formula was used:

$$y_{ik} = \alpha_k + \delta T_{ik} + \beta x_{ik} + \gamma x_{ik} T_{ik} + \varepsilon_{ik}$$

Where:

- y_{ik} is the PHQ-9 post-test score of individual i in trial k;
- α_k is the trial-specific ("stratified") intercept;
- T_{ik} is the treatment assignment of individual i in trial k (0 = control, 1 = intervention), and δ the average treatment effect;
- x_{ik} is the value of individual i in trial k on the tested baseline covariate x, with β being the associated regression weight;
- γ captures the treatment-covariate interaction (i.e., moderation) of δ for varying values of x;
- ε_{ik} is the prediction error of individual i in trial k.

The model was fitted using OLS regression (lm function in R). To guard against misspecification and account for the nested data structure (patients in trials), cluster-robust variance estimation was employed for *P*-values and confidence intervals, using the CR2 estimator by Pustejovsky & Tipton (2018).

References

Chung Y, Rabe-Hesketh S, Dorie V, Gelman A, Liu J (2013). "A nondegenerate penalized likelihood estimator for variance parameters in multilevel models." *Psychometrika* 78 (4), 685-709. DOI: 10.1007/s11336-013-9328-2.

Pustejovsky, J. E., & Tipton, E. (2018). Small-sample methods for cluster-robust variance estimation and hypothesis testing in fixed effects models. *Journal of Business & Economic Statistics*, *36*(4), 672-683.

S5. Risk of Bias summary.

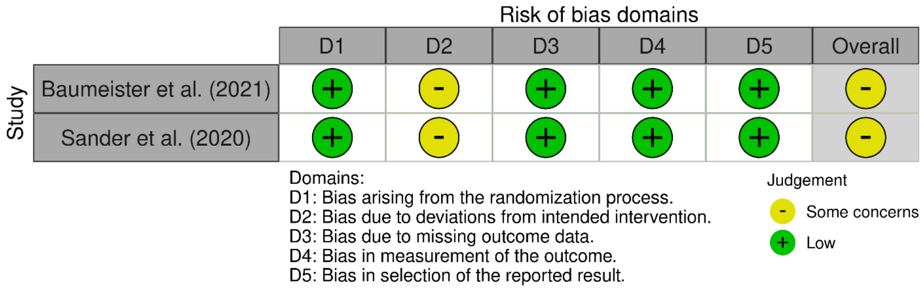

S6. Effects on reliable change.

RCI	Control (n= 251)			Intervention (n= 253)		
	Deteriorated	Unchanged	Improved	Deteriorated	Unchanged	Improved
n	14	209	28	5	186	62
%	5.58%	83.27%	11.16%	1.98%	73.52%	24.52%

Note. Based on the imputed data. RCI values were determined using the standard deviation of the baseline PHQ-9 value and assuming a reliability of r_{yy}=0.84 (Kroenke et al., 2001). The χ^2 test showed that significantly more participants of the intervention group experienced a reliable improvement compared to the control group: $F_{1,1515.47} = 12.36$, $p < .001$.

References:

Kroenke, K., Spitzer, R. L., & Williams, J. B. (2001). The PHQ-9: validity of a brief depression severity measure. *Journal of General Internal Medicine*, *16*(9), 606-613.

S7. Regression coefficients of univariate moderator analyses (main model; complete cases only).

Baseline Variable	$\hat{\beta}$	$\sqrt{\hat{V}_\beta}$	t	$p(<\|t\|)$	$\hat{\tau}^2_{\text{Intercept}}$	$\hat{\tau}^2_{\text{Slope}}$
**Socio-demographics**						
Age	0.010	0.477	0.022	0.983	1.471	0.719
Gender, female	-1.329	0.939	-1.416	0.158	1.476	0.716
Relationship, yes	-1.328	0.938	-1.616	0.158	1.472	0.722

Education, >13 years	-0.659	0.954	-0.690	0.490	1.450	0.733
Children, yes	-0.887	0.939	-0.944	0.346	1.464	0.713
Lifetime SCID diagnosis, yes	-0.380	1.453	-0.262	0.794	0.941	0.920
Previous psychotherapy, yes	0.180	0.939	0.192	0.848	1.372	0.701
Previous depression medication, yes	0.716	0.937	0.765	0.445	1.412	0.717
Previous back pain medication, yes	-1.981	0.934	-2.121	0.035	1.467	0.707
Sick leave, yes	-1.143	0.994	-1.150	0.251	1.314	0.691
Social support, (very) good	1.891	0.935	0.953	0.341	1.444	0.717
Internet affinity (IAS)	-0.022	0.473	-0.047	0.963	1.450	0.718
Recruitment, online	1.523	0.941	1.618	0.106	1.503	0.734
**Symptom severity**						
Depressive symptom severity (PHQ-9)	-0.771	0.395	-1.951	0.052	0.626	0.564
Depressive symptom severity (HAM-D-17)	-0.580	0.481	-1.205	0.229	0.597	0.686
Pain intensity (NRS)	-0.379	0.465	-0.817	0.414	1.396	0.720
Quality of life (AQoL-6D)	-0.436	0.431	-1.012	0.312	0.641	0.647
**Pain-related risk factors**						
Pain self-efficacy (PSEQ)	0.204	0.457	0.446	0.656	1.180	0.827
Subjective employment forecast (SPE)	-0.570	0.470	1.211	0.227	1.379	0.720
Pain-related disability (ODI)	-0.220	0.453	-0.486	0.627	1.220	0.729

Note. A Wishart prior was used for the covariance-variance matrix of the random effects. AQoL-6D: Assessment of Quality of Life, HAM-D-17: Hamilton Depression Rating Scale, IAS: Internet Affinity Scale; NRS: numerical rating scale, ODI: Oswestry Disability Scale, PHQ-9: Patient Health Questionnaire, PSEQ: Pain self-efficacy questionnaire, SPE: Subjective Prognostic Employment Scale.

S8. Regression coefficients of univariate moderator analyses (OLS regression).

Baseline Variable	Treatment-Covariate Interaction			
	$\hat{\beta}$	$\sqrt{\hat{V}_{\beta}}$	t	$p(<\|t\|)$
**Socio-demographics**				
Age	0.119	0.452	0.263	0.792
Gender, female	-0.688	0.919	-0.749	0.453
Relationship, yes	-0.831	0.879	-0.945	0.343
Education, >13 years	-0.354	0.910	-0.389	0.696

Children, yes	-0.687	0.894	-0.768	0.441
Lifetime SCID diagnosis, yes	-1.067	0.906	-1.178	0.238
Previous psychotherapy, yes	-0.179	0.917	-0.195	0.844
Previous depression medication, yes	0.303	0.920	0.329	0.741
Previous back pain medication, yes	-2.020	0.892	-2.265	0.024
Sick leave, yes	-1.422	1.012	-1.405	0.159
Social support, (very) good	1.107	0.891	1.242	0.214
Internet affinity (IAS)	-0.025	0.466	-0.054	0.956
Recruitment, online	0.904	0.886	1.020	0.307
**Symptom severity**				
Depressive symptom severity (PHQ-9)	-0.799	0.377	-2.119	0.035
Depressive symptom severity (HAM-D-17)	-0.816	0.424	-1.924	0.054
Pain intensity (NRS)	-0.510	0.442	-1.069	0.248
Quality of life (AQoL-6D)	-0.636	0.398	-1.598	0.112
**Pain-related risk factors**				
Pain self-efficacy (PSEQ)	0.357	0.423	0.844	0.398
Subjective employment forecast (SPE)	-0.385	0.447	-0.861	0.388
Pain-related disability (ODI)	-0.409	0.421	-0.971	0.330

Note. P-values based on cluster-robust variance estimation (CR2 estimator). AQoL-6D: Assessment of Quality of Life, HAM-D-17: Hamilton Depression Rating Scale, IAS: Internet Affinity Scale; NRS: numerical rating scale, ODI: Oswestry Disability Scale, PHQ-9: Patient Health Questionnaire, PSEQ: Pain self-efficacy questionnaire, SPE: Subjective Prognostic Employment Scale.

S9. Regression coefficients of univariate moderator analyses (OLS regression; complete cases).

Baseline Variable	Treatment-Covariate Interaction			
	$\hat{\beta}$	$\sqrt{\hat{V}_{\beta}}$	t	$p(<\lvert t\rvert)$
**Socio-demographics**				
Age	0.008	0.475	0.016	0.987
Gender, female	-1.333	0.938	-2.029	0.156
Relationship, yes	-1.304	0.936	-1.392	0.165
Education, >13 years	-0.556	0.943	-0.589	0.556
Children, yes	-0.882	0.937	-0.941	0.347
Lifetime SCID diagnosis, yes	-0.558	0.989	-0.565	0.573

Previous psychotherapy, yes	0.141	0.927	0.152	0.880
Previous depression medication, yes	0.618	0.926	0.667	0.505
Previous back pain medication, yes	-2.000	0.933	-2.143	0.033
Sick leave, yes	-1.148	0.989	-1.160	0.246
Social support, (very) good	0.958	0.931	1.029	0.304
Internet affinity (IAS)	-0.041	0.470	-0.087	0.931
Recruitment, online	1.447	0.936	1.546	0.123
**Symptom severity**				
Depressive symptom severity (PHQ-9)	-0.765	0.365	-2.095	0.037
Depressive symptom severity (HAM-D-17)	-0.610	0.432	-1.412	0.159
Pain intensity (NRS)	-0.431	0.461	-0.934	0.351
Quality of life (AQoL-6D)	-0.502	0.405	-1.240	0.216
**Pain-related risk factors**				
Pain self-efficacy (PSEQ)	0.404	0.443	0.912	0.363
Subjective employment forecast (SPE)	-0.612	0.467	-1.319	0.188
Pain-related disability (ODI)	-0.320	0.444	-0.722	0.471

Note. AQoL-6D: Assessment of Quality of Life, HAM-D-17: Hamilton Depression Rating Scale, IAS: Internet Affinity Scale; NRS: numerical rating scale, ODI: Oswestry Disability Scale, PHQ-9: Patient Health Questionnaire, PSEQ: Pain self-efficacy questionnaire, SPE: Subjective Prognostic Employment Scale.

S10. Moderator-specific marginal means and change scores.

		PHQ-9 Score					Test of Moderation (Change Score Analysis)	
		Baseline (*M*, *SE*)	Post-Test (*M*, *SE*)	Change (*M*, *SE*)	Δ Change (*M*, *SE*)	Diff. Δ Change (*M*, *SE*)	*F/t*	*p*
**Age, Percentiles**								
< 20th	Control	10.53 (0.570)	9.69 (0.710)	-0.84 (0.592)			0.45	0.772
	Intervention	10.96 (0.508)	7.47 (0.653)	-3.49 (0.550)	-2.65 (0.825)	Ref.		
< 40th	Control	10.49 (0.566)	9.53 (0.717)	-0.96 (0.600)				
	Intervention	10.61 (0.498)	7.58 (0.622)	-3.03 (0.524)	-2.06 (0.799)	-0.59 (1.130), *n.s.*		
40th to 60th	Control	10.89 (0.537)	9.62 (0.680)	-1.27 (0.579)				
	Intervention	10.19 (0.575)	7.64 (0.765)	-2.55 (0.655)	-1.28 (0.881)	-1.37 (1.182), *n.s.*		
> 60th	Control	10.79 (0.545)	10.07 (0.726)	-0.72 (0.627)				
	Intervention	9.92 (0.635)	6.97 (0.818)	-2.95 (0.689)	-2.23 (0.924)	-0.42 (1.216), *n.s.*		
> 80th	Control	10.02 (0.571)	8.97 (0.706)	-1.04 (0.596)				

		PHQ-9 Score					Test of Moderation (Change Score Analysis)	
		Baseline (*M*, *SE*)	Post-Test (*M*, *SE*)	Change (*M*, *SE*)	Δ Change (*M*, *SE*)	Diff. Δ Change (*M*, *SE*)	*F/t*	*p*
	Intervention	9.90 (0.576)	7.39 (0.766)	-2.50 (0.654)	-1.46 (0.865)	-1.19 (1.171), *n.s.*		
**Gender**								
Female	Control	10.63 (0.312)	9.46 (0.393)	-1.17 (0.336)			0.10	0.748
	Intervention	10.65 (0.321)	7.60 (0.413)	-3.05 (0.349)	-1.89 (0.495)	Ref.		
Male	Control	10.44 (0.410)	9.80 (0.525)	-0.64 (0.444)				
	Intervention	10.01 (0.385)	7.23 (0.534)	-2.78 (0.465)	-2.15 (0.660)	-0.26 (0.809), *n.s.*		
**Relationship**								
No	Control	10.89 (0.480)	9.76 (0.613)	-1.13 (0.515)			0.02	0.898
	Intervention	11.29 (0.455)	8.28 (0.590)	-3.02 (0.500)	-1.89 (0.720)	Ref.		
Yes	Control	10.44 (0.289)	9.52 (0.368)	-0.92 (0.315)				
	Intervention	10.01 (0.292)	7.11 (0.387)	-2.91 (0.332)	-1.99 (0.461)	-0.10 (0.816), *n.s.*		
**Education**								
$\leq$ 13 years	Control	10.52 (0.265)	9.52 (0.340)	-1.00 (0.291)			0.20	0.651
	Intervention	10.48 (0.263)	7.44 (0.350)	-3.04 (0.301)	-2.03 (0.427)	Ref.		
$>$ 13 years	Control	10.82 (0.712)	10.11 (0.880)	-0.72 (0.733)				
	Intervention	9.73 (0.699)	7.50 (0.973)	-2.23 (0.843)	-1.51 (1.091)	0.52 (1.150), *n.s.*		
**Children**								
No	Control	10.34 (0.562)	8.98 (0.733)	-1.36 (0.622)			0.90	0.344
	Intervention	10.14 (0.539)	7.53 (0.693)	-2.61 (0.586)	-1.26 (0.860)	Ref.		
Yes	Control	10.61 (0.277)	9.73 (0.356)	-0.88 (0.304)				
	Intervention	10.45 (0.278)	7.42 (0.377)	-3.03 (0.326)	-2.15 (0.445)	-0.89 (0.942), *n.s.*		
**Lifetime SCID diagnosis**								
No	Control	9.66 (0.711)	8.72 (0.895)	-0.94 (0.753)			1.11	0.293
	Intervention	9.61 (0.709)	7.05 (0.923)	-2.56 (0.786)	-1.62 (0.525)	Ref.		
Yes	Control	11.42 (0.683)	10.48 (0.872)	-0.94 (0.735)				
	Intervention	11.10 (0.683)	7.73 (0.866)	-3.36 (0.727)	-2.42 (0.593)	-0.81 (0.768), *n.s.*		
**Previous psychotherapy**								
No	Control	10.06 (0.316)	9.37 (0.416)	-0.68 (0.357)			0.32	0.574
	Intervention	9.80 (0.309)	7.31 (0.416)	-2.49 (0.354)	-1.81 (0.505)	Ref.		
Yes	Control	11.33 (0.401)	9.92 (0.518)	-1.42 (0.447)				
	Intervention	11.35 (0.403)	7.66 (0.561)	-3.68 (0.486)	-2.27 (0.651)	-0.46 (0.816), *n.s.*		
**Previous depression medication**								
No	Control	10.33 (0.296)	9.48 (0.392)	-0.85 (0.336)			0.00	0.985
	Intervention	10.05 (0.298)	7.24 (0.398)	-2.81 (0.342)	-1.97 (0.472)	Ref.		
Yes	Control	11.08 (0.465)	9.81 (0.604)	-1.27 (0.512)				
	Intervention	11.12 (0.447)	7.90 (0.606)	-3.22 (0.523)	1.95 (0.731)	0.02 (0.853), *n.s.*		

		PHQ-9 Score					Test of Moderation (Change Score Analysis)	
		Baseline (*M*, *SE*)	Post-Test (*M*, *SE*)	Change (*M*, *SE*)	Δ Change (*M*, *SE*)	Diff. Δ Change (*M*, *SE*)	*F/t*	*p*
**Previous back pain medication**								
No	Control	9.46 (0.405)	7.91 (0.501)	-1.55 (0.426)			6.606	0.010
	Intervention	9.32 (0.412)	7.11 (0.544)	-2.21 (0.467)	-0.65 (0.644)	Ref.		
Yes	Control	11.18 (0.305)	10.53 (0.393)	-0.64 (0.340)				
	Intervention	10.97 (0.302)	7.63 (0.413)	-3.24 (0.361)	-2.70 (0.500)	-2.05 (0.797)*		
**Sick leave**								
No	Control	9.18 (0.458)	8.17 (0.600)	-1.01 (0.512)			0.01	0.935
	Intervention	10.06 (0.464)	7.03 (0.609)	-3.02 (0.522)	-2.01 (0.730)	Ref.		
Yes	Control	11.23 (0.307)	10.27 (0.399)	-0.96 (0.336)				
	Intervention	10.53 (0.309)	7.63 (0.413)	-2.90 (0.355)	-1.94 (0.503)	0.07 (0.906), *n.s.*		
**Social support**								
< good	Control	11.30 (0.316)	10.29 (0.409)	-1.01 (0.351)			0.51	0.474
	Intervention	10.86 (0.306)	7.67 (0.423)	-3.19 (0.368)	-2.18 (0.524)	Ref.		
≥ good	Control	9.44 (0.381)	8.54 (0.493)	-0.90 (0.419)				
	Intervention	9.58 (0.395)	7.05 (0.504)	-2.52 (0.427)	-1.62 (0.597)	0.56 (0.775), *n.s.*		
**Internet affinity (IAS), *Percentiles***								
< 20th	Control	10.42 (0.521)	8.86 (0.660)	-1.56 (0.567)			0.24	0.915
	Intervention	9.77 (0.562)	6.65 (0.685)	-3.12 (0.568)	-1.57 (0.803)	Ref.		
< 40th	Control	11.42 (0.553)	10.44 (0.680)	-0.98 (0.571)				
	Intervention	10.54 (0.520)	7.98 (0.666)	-2.55 (0.563)	-1.57 (0.809)	-0.01 (1.133), *n.s.*		
40th to 60th	Control	10.20 (0.555)	9.52 (0.702)	-0.68 (0.597)				
	Intervention	10.77 (0.602)	7.77 (0.785)	-2.99 (0.668)	-2.31 (0.896)	-0.75 (1.190), *n.s.*		
> 60th	Control	9.73 (0.544)	8.77 (0.701)	-0.96 (0.598)				
	Intervention	10.14 (0.520)	7.02 (0.670)	-3.12 (0.573)	-2.16 (0.841)	-0.60 (1.126), *n.s.*		
> 80th	Control	11.17 (0.607)	10.62 (0.764)	-0.55 (0.643)				
	Intervention	10.75 (0.558)	7.77 (0.743)	-2.98 (0.653)	-2.43 (0.906)	-0.87 (1.237), *n.s.*		
**Recruitment**								
Offline	Control	10.67 (0.300)	9.88 (0.380)	-0.80 (0.323)			0.01	0.921
	Intervention	10.24 (0.294)	7.44 (0.402)	-2.80 (0.350)	-2.00 (0.472)	Ref.		
Online	Control	10.29 (0.446)	8.93 (0.578)	-1.36 (0.489)				
	Intervention	10.76 (0.459)	7.48 (0.580)	-3.28 (0.486)	-1.92 (0.697)	0.08 (0.812) , *n.s.*		
**Depressive symptom severity (PHQ-9), *Percentiles***								
< 20th	Control	4.09 (0.211)	4.77 (0.625)	0.68 (0.601)			1.18	0.319
	Intervention	4.61 (0.201)	3.82 (0.635)	-0.79 (0.61)	-1.46 (0.805)	Ref.		
< 40th	Control	7.81 (0.311)	7.03 (0.626)	-0.78 (0.556)				
	Intervention	7.74 (0.294)	5.57 (0.609)	-2.17 (0.525)	-1.38 (0.761)	0.08 (1.084), *n.s.*		
40th to 60th	Control	10.32 (0.321)	9.22 (0.610)	-1.1 (0.554)				
	Intervention	10.25 (0.315)	7.75 (0.684)	-2.5 (0.681)	-1.40 (0.861)	0.07 (1.158), *n.s.*		
> 60th	Control	12.47 (0.23)	11.38 (0.706)	-1.09 (0.675)				
	Intervention	12.38 (0.219)	8.41 (0.757)	-3.97 (0.732)	-2.88 (1.013)	-1.41 (1.283), *n.s.*		
> 80th	Control	17.02 (0.217)	14.92 (0.635)	-2.1 (0.602)				
	Intervention	16.63 (0.216)	11.26 (0.619)	-5.37 (0.591)	-3.26 (0.823)	-1.80 (1.160), *n.s.*		

		PHQ-9 Score					Test of Moderation (Change Score Analysis)	
		Baseline (*M*, *SE*)	Post-Test (*M*, *SE*)	Change (*M*, *SE*)	Δ Change (*M*, *SE*)	Diff. Δ Change (*M*, *SE*)	*F/t*	*p*
**Depressive symptom severity (HAM-D-17),** ***Percentiles***								
< 20th	Control	6.87 (0.510)	6.31 (0.671)	-0.56 (0.608)	·		0.48	0.747
	Intervention	6.98 (0.479)	4.69 (0.644)	-2.29 (0.577)	-1.73 (0.784)	Ref.		
< 40th	Control	9.37 (0.536)	7.82 (0.699)	-1.55 (0.620)				
	Intervention	9.61 (0.486)	6.55 (0.670)	-3.06 (0.609)	-1.51 (0.850)	0.22 (1.140), *n.s.*		
40th to 60th	Control	10.14 (0.463)	8.78 (0.616)	-1.36 (0.549)				
	Intervention	10.56 (0.547)	7.51 (0.744)	-3.04 (0.680)	-1.69 (0.865)	0.05 (1.185), *n.s.*		
> 60th	Control	11.85 (0.475)	10.74 (0.632)	-1.11 (0.568)				
	Intervention	11.57 (0.474)	8.36 (0.642)	-3.2 (0.578)	-2.09 (0.824)	-0.35 (1.115), *n.s.*		
> 80th	Control	14.08 (0.567)	13.91 (0.772)	-0.16 (0.700)				
	Intervention	13.06 (0.544)	9.83 (0.840)	-3.23 (0.775)	-3.07 (0.986)	-1.33 (1.249), *n.s.*		
**Pain intensity (NRS)**								
Rated < 1	Control	7.51 (1.133)	6.25 (1.456)	-1.26 (1.283)	.		0.31	0.816
	Intervention	8.98 (1.204)	6.77 (1.768)	-2.22 (1.578)	-0.96 (1.999)	Ref.		
Rated < 2	Control	9.13 (0.425)	8.37 (0.545)	-0.76 (0.471)				
	Intervention	9.14 (0.411)	6.55 (0.539)	-2.59 (0.464)	-1.83 (0.654)	-0.87 (0.861), *n.s.*		
Rated < 3	Control	11.11 (0.313)	9.97 (0.413)	-1.13 (0.359)				
	Intervention	10.54 (0.322)	7.45 (0.458)	-3.09 (0.408)	-1.96 (0.559)	-1.00 (1.359), *n.s.*		
Rated < 4	Control	13.26 (0.800)	12.74 (1.006)	-0.51 (0.869)				
	Intervention	13.50 (0.706)	10.05 (0.949)	-3.45 (0.835)	-2.94 (1.188)	-1.98 (1.302), *n.s.*		
**Quality of life (AQoL-6D),** ***Percentiles***								
< 20th	Control	6.25 (0.438)	5.43 (0.609)	-0.81 (0.580)			0.78	0.537
	Intervention	6.84 (0.413)	4.35 (0.622)	-2.49 (0.591)	-1.67 (0.788)	Ref.		
< 40th	Control	9.55 (0.403)	8.35 (0.555)	-1.19 (0.531)				
	Intervention	8.25 (0.474)	5.55 (0.667)	-2.70 (0.628)	-1.51 (0.814)	0.17 (1.107), *n.s.*		
40th to 60th	Control	10.44 (0.487)	9.74 (0.717)	-0.70 (0.679)				
	Intervention	10.14 (0.430)	7.72 (0.616)	-2.42 (0.583)	-1.72 (0.908)	-0.05 (1.148), *n.s.*		
> 60th	Control	12.05 (0.446)	10.31 (0.634)	-1.73 (0.598)				
	Intervention	11.51 (0.478)	7.93 (0.718)	-3.57 (0.692)	-1.84 (0.922)	-0.17 (1.198), *n.s.*		
> 80th	Control	14.40 (0.460)	14.15 (0.658)	-0.24 (0.620)				
	Intervention	14.33 (0.424)	10.77 (0.641)	-3.56 (0.610)	-3.32 (0.853)	-1.64 (1.169), *n.s.*		
**Pain self-efficacy (PSEQ),** ***Percentiles***								
< 20th	Control	12.58 (0.537)	12.17 (0.703)	-0.41 (0.622)			0.98	0.415
	Intervention	12.84 (0.497)	9.81 (0.644)	-3.03 (0.569)	-2.61 (0.855)	Ref.		
< 40th	Control	11.24 (0.491)	10.74 (0.631)	-0.50 (0.560)				
	Intervention	11.20 (0.520)	7.83 (0.692)	-3.37 (0.626)	-2.87 (0.850)	-0.25 (1.185), *n.s.*		
40th to 60th	Control	10.62 (0.495)	9.48 (0.634)	-1.14 (0.558)				
	Intervention	9.44 (0.531)	6.27 (0.743)	-3.16 (0.675)	-2.02 (0.867)	0.60 (1.161), *n.s.*		
> 60th	Control	8.99 (0.543)	7.93 (0.677)	-1.05 (0.595)				
	Intervention	9.65 (0.543)	7.20 (0.724)	-2.46 (0.645)	-1.40 (0.859)	1.21 (1.195), *n.s.*		
> 80th	Control	8.82 (0.540)	7.01 (0.686)	-1.80 (0.615)				
	Intervention	8.07 (0.516)	5.39 (0.666)	-2.68 (0.587)	-0.88 (0.827)	1.74 (1.170), *n.s.*		
**Subjective employment forecast (SPE)**								
Rated 1	Control	9.03 (0.477)	7.44 (0.607)	-1.59 (0.525)			1.45	0.226
	Intervention	8.95 (0.498)	6.43 (0.659)	-2.53 (0.579)	-0.94 (0.771)	Ref.		

		PHQ-9 Score					Test of Moderation (Change Score Analysis)	
		Baseline (*M*, *SE*)	Post-Test (*M*, *SE*)	Change (*M*, *SE*)	Δ Change (*M*, *SE*)	Diff. Δ Change (*M*, *SE*)	*F/t*	*p*
Rated 2	Control	9.93 (0.591)	9.60 (0.751)	-0.33 (0.644)				
	Intervention	9.51 (0.498)	6.34 (0.637)	-3.17 (0.541)	-2.84 (0.832)	-1.90 (1.128), *n.s.*		
Rated 3	Control	11.04 (0.416)	9.95 (0.528)	-1.09 (0.467)				
	Intervention	10.76 (0.431)	8.07 (0.587)	-2.68 (0.508)	-1.59 (0.715)	-0.65 (1.019), *n.s.*		
Rated 4	Control	11.80 (0.480)	11.18 (0.624)	-0.62 (0.539)				
	Intervention	12.07 (0.497)	8.59 (0.690)	-3.48 (0.611)	-2.86 (0.828)	-1.925 (1.134), *n.s.*		
**Pain-related disability (ODI), *Percentiles***								
< 20th	Control	8.78 (0.472)	7.62 (0.605)	-1.16 (0.543)			0.75	0.555
	Intervention	8.05 (0.471)	5.09 (0.624)	-2.96 (0.555)	-1.80 (0.754)	Ref.		
< 40th	Control	8.56 (0.549)	7.44 (0.710)	-1.12 (0.636)				
	Intervention	9.41 (0.550)	6.42 (0.724)	-2.99 (0.652)	-1.87 (0.916)	-0.07 (1.195), *n.s.*		
40th to 60th	Control	11.15 (0.480)	10.01 (0.640)	-1.13 (0.574)				
	Intervention	10.16 (0.549)	8.14 (0.757)	-2.02 (0.692)	-0.89 (0.933)	0.91 (1.184), *n.s.*		
> 60th	Control	10.87 (0.514)	10.56 (0.659)	-0.31 (0.583)				
	Intervention	10.81 (0.502)	7.56 (0.703)	-3.24 (0.639)	-2.93 (0.858)	-1.14 (1.119), *n.s.*		
> 80th	Control	13.45 (0.534)	12.39 (0.727)	-1.07 (0.650)				
	Intervention	12.99 (0.481)	9.68 (0.654)	-3.31 (0.592)	-2.24 (0.872)	-0.44 (1.142), *n.s.*		

*$p<0.05$, **$p<0.01$, *** $p<0.001$.

Note: Change Score Analysis was based on a linear OLS regression with stratification terms for trial; see "OLS Regression Model" formula in S4. Change scores were used as the response, and percentile groups/categorical predictors were used as the moderator variable.

S11. PHQ-9 symptom changes in baseline PHQ-9 percentile groups.

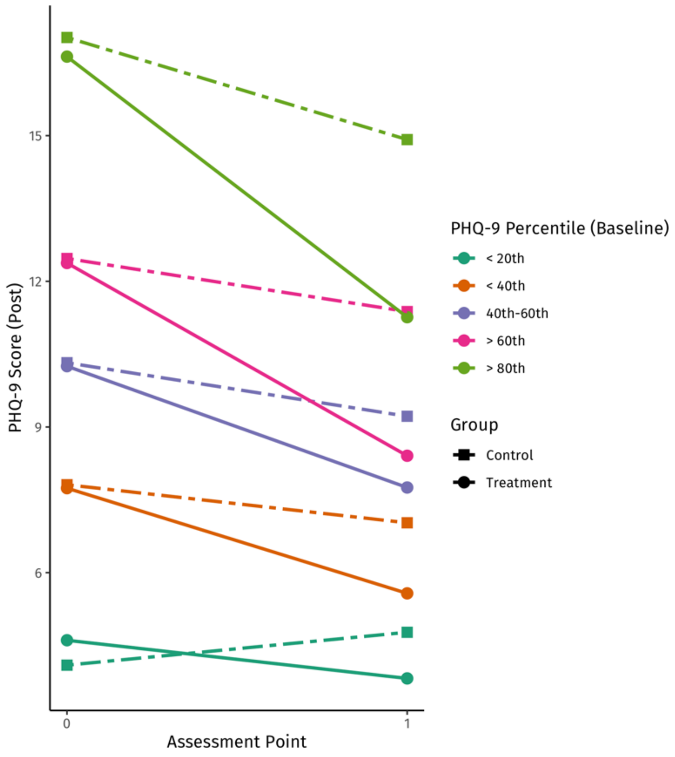

S12. Correlation matrix of moderator variables.

	Sex	Age	Relationship Status	Children	Education	Previous Depression Medication	Previous Psychotherapy	Social Support	Internet Affinity	Subjective Employment Forecast	Recruitment	Sick Leave	Previous Back Pain Medication	Lifetime SCID Diagnosis	Quality of Life	Depressive Symptoms (HAM-D-17)	Depressive Symptoms (PHQ-9)	Pain-related Disability	Pain Intensity	Pain Self-Efficacy
Sex	-0.01 (0.047)	0.045	0.044	0.045	0.043	0.045	0.044	0.045	0.044	0.045	0.045	0.05	0.045	0.045	0.045	0.045	0.045	0.045	0.045	0.045
Age	-0,03	-0.04 (0.048)	0.043	0.041	0.044	0.045	0.045	0.045	0.044	0.045	0.045	0.056	0.044	0.044	0.045	0.044	0.044	0.045	0.045	0.045
Relationship Status	-0,07	0,20	0.03 (0.047)	0.04	0.044	0.045	0.045	0.045	0.044	0.045	0.045	0.051	0.045	0.045	0.045	0.045	0.045	0.045	0.044	0.045
Children	-0,01	0,29	0,31	0.04 (0.049)	0.044	0.045	0.044	0.045	0.045	0.046	0.044	0.047	0.045	0.045	0.045	0.045	0.045	0.045	0.045	0.045
Education	-0,19	0,08	-0,08	-0,13	-0.13 (0.048)	0.045	0.045	0.043	0.045	0.044	0.044	0.046	0.044	0.038	0.041	0.042	0.042	0.044	0.043	0.042
Previous Depression Medication	-0,09	0,05	0,02	0,06	-0,08	0.09 (0.05)	0.036	0.045	0.045	0.045	0.045	0.051	0.045	0.043	0.043	0.044	0.044	0.045	0.045	0.045
Previous Psychotherapy	-0,13	0,01	0,05	0,11	-0,02	0,44	0.11 (0.049)	0.045	0.045	0.045	0.045	0.049	0.045	0.043	0.043	0.042	0.042	0.045	0.045	0.045
Social Support	-0,03	-0,03	-0,06	-0,05	0,16	-0,07	-0,03	-0.21 (0.045)	0.044	0.044	0.044	0.047	0.045	0.044	0.041	0.042	0.041	0.043	0.044	0.043
Internet Affinity	0,14	-0,12	-0,06	-0,06	0.00	0,06	0,04	-0,10	0.10 (0.048)	0.045	0.045	0.048	0.045	0.044	0.044	0.044	0.044	0.044	0.045	0.044
Subjective Employment Forecast	0,09	0,15	0,07	0,06	-0,14	0,09	0.00	-0,16	0,01	0.26 (0.044)	0.045	0.048	0.044	0.044	0.038	0.044	0.041	0.038	0.041	0.038
Recruitment	-0,06	0,05	-0,03	-0,12	0,09	0,07	0,01	0,06	0,06	0,06	0.00 (0.048)	0.049	0.045	0.044	0.044	0.044	0.045	0.045	0.045	0.045
Sick Leave	-0,01	-0,07	-0,02	-0,08	-0,10	-0,02	0,03	-0,08	0,07	0,15	-0,01	0.16 (0.051)	0.048	0.049	0.049	0.048	0.048	0.049	0.049	0.049
Previous Back Pain Medication	-0,05	-0,08	0.00	-0,04	-0,08	0,03	-0,03	-0,03	-0,01	0,13	-0,05	0,14	0.17 (0.047)	0.045	0.042	0.044	0.043	0.039	0.042	0.041
Lifetime SCID Diagnosis	0,02	-0,16	0,01	0,02	-0,38	0,19	0,22	-0,15	0,13	0,13	0,09	0,11	0,05	0.28 (0.046)	0.035	0.03	0.033	0.041	0.044	0.04
Quality of Life	0,03	-0,06	0,05	0,06	-0,28	0,19	0,20	-0,30	0,15	0,40	0,07	0,24	0,25	0,46	0.54 (0.035)	0.03	0.02	0.022	0.034	0.023
Depressive Symptoms (HAM-D-17)	-0,03	-0,09	0,02	0,02	-0,25	0,17	0,26	-0,23	0,09	0,19	0,09	0,16	0,12	0,58	0,56	0.46 (0.04)	0.026	0.037	0.042	0.038
Depressive Symptoms (PHQ-9)	-0,03	-0,13	0,02	0,04	-0,27	0,18	0,26	-0,30	0,10	0,31	0,07	0,20	0,21	0,52	0,74	0,66	0.63 (0.029)	0.033	0.039	0.035
Pain-related Disability	0,02	-0,02	0,04	0,01	-0,14	0,05	0.00	-0,18	0,10	0,40	0,03	0,23	0,35	0,29	0,71	0,42	0,52	0.39 (0.041)	0.031	0.023
Pain Intensity	0,05	0,01	0,08	0,06	-0,21	0,01	0,03	-0,08	-0,02	0,31	0,02	0,18	0,28	0,16	0,49	0,26	0,36	0,56	0.26 (0.046)	0.034
Pain Self-Efficacy	-0,03	0,03	-0,01	0	0,23	-0,11	-0,08	0,22	-0,09	-0,40	-0,05	-0,20	-0,30	-0,33	-0,69	-0,40	-0,48	-0,70	-0,50	-0.39 (0.04)

Note. The lower triangle shows the correlation between moderator variables, and the upper triangle the corresponding standard errors. The diagonal elements show the correlation of the moderator variable with the target outcome (PHQ-9 at post-test), with the standard error in brackets.

S13. Variable permutation importance (model-based random forest).

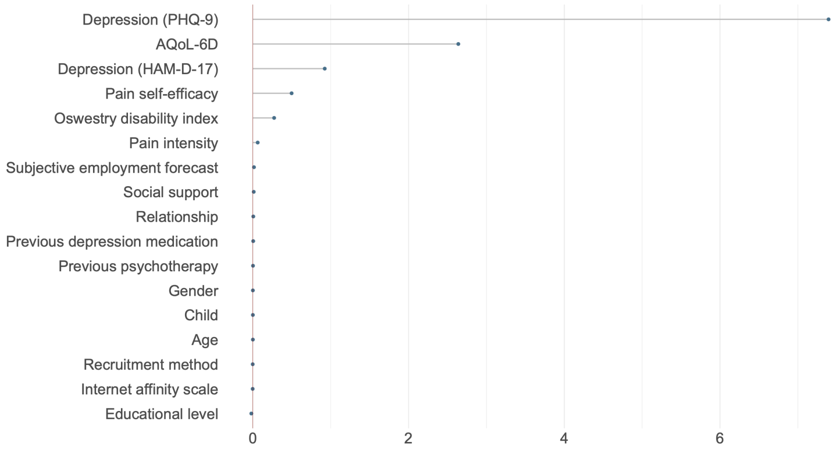

S14. Variable importances (complete cases only).

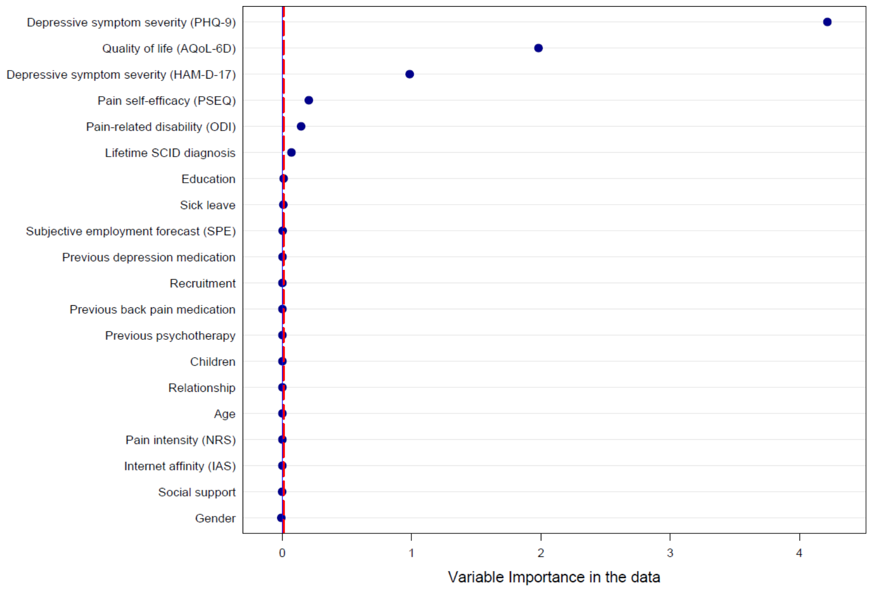

Note: Pain Intensity (NRS), Internet Affinity (IAS), Social Support and Gender had negative variable importance scores.

S15. Model-based recursive partitioning (complete cases only; *n*=382).

Node	Cohen's *d* (95% CI)	S.E.	*n*	n_{IG}	n_{CG}
PHQ-9 ≤ 6	-0.383 [-0.837; 0.072]	0.232	77	40	37
6 < PHQ-9 ≤ 10 & AQoL-6D ≤ 43	-0.501 [-1.151; 0.150]	0.332	39	18	21
6 < PHQ-9 ≤ 10 & AQoL-6D > 43	-0.504 [-0.903; -0.105]	0.204	99	51	48
10 < PHQ-9 ≤ 15 & PSEQ ≤ 38	-1.278 [-1.726; -0.839]	0.229	79	39	40
10 < PHQ-9 ≤ 15 & PSEQ > 38	0.218 [-0.495; 0.931]	0.364	37	12	25
PHQ-9 > 15	-0.478 [-1.041; 0.086]	0.288	51	24	27

S16. Reliable change in terminal tree nodes.

Node	*RCI*	Control			Intervention			χ^2 Test[a]
		Deteriorated	Unchanged	Improved	Deteriorated	Unchanged	Improved	
PHQ-9 ≤ 6	*n*	19	22	9	9	29	17	$F_{1,1085.2}$=1.062
	%	38%	44%	18%	16.36%	52.73%	30.91%	p=0.303
PHQ-9 ≤ 10,	*n*	2	8	12	2	3	22	$F_{1,\ 1837.66}$=2.390
PHQ-9 > 6,	%	9.09%	36.36%	54.55%	7.41%	11.11%	81.48%	p=0.122
AQOL-6D ≤ 43								
PHQ-9 ≤ 10,	*n*	18	18	24	9	18	35	$F_{1,2340.4}$=2.163
PHQ-9 > 6,	%	30%	30%	40%	14.52%	29.03%	56.45%	p=0.141
AQOL-6D > 43								
PHQ-9 ≤ 15,	*n*	16	17	18	3	12	45	$F_{1,681.39}$=11.729
PHQ-9 > 10,	%	31.37%	33.33%	35.29%	5%	20%	75%	p<0.001
PSEQ ≤ 38								
PHQ-9 ≤ 15,	n	3	6	17	3	4	10	$F_{1,47174.22}$=0.006,
PHQ-9 > 10,	%	11.54%	23.08%	65.38%	17.65%	23.53%	58.82%	p=0.936
PSEQ > 38								
PHQ-9 > 15	n	3	13	19	1	7	21	$F_{1,2130.7}$=1.194
	%	8.57%	37.14%	54.29%	3.45%	24.14%	72.41%	p=0.274

[a]Test for differences in reliable improvement between groups

S17. Non-zero parameters of the linear mixed model with ℓ_1-estimation (glmmLasso).

Prognostic Predictors

	Intercept	Group	PHQ-9	AQOL-6D	HAM-D-17	PSEQ	ODI
β	4.731***	-0.393	0.558	0.065	0.083	-0.053	-0.006

Prescriptive Predictor

	PSEQ	Education[a]
β	0.036***	1.291

Variance component

	Intercept	Slope
τ	0.285	0.095

Note. Values represent unstandardized regression weights. Penality parameter λ=670 determined based on the BIC.
[a]up to high school only, *yes*
***p<0.001
